# Non-adherence to anti-hypertensive medications in a low-resource country Nepal: a systematic review and meta-analysis

**DOI:** 10.1097/MS9.0000000000001088

**Published:** 2023-07-19

**Authors:** Pashupati Pokharel, Saroj Kumar Jha, Alisha Adhikari, Srijana Katwal, Sagun Ghimire, Abhigan Babu Shrestha, Nahakul Poudel

**Affiliations:** a Maharajgunj Medical Campus, Institute of Medicine, Tribhuvan University; b Shree Birendra Hospital, Chhauni, Kathmandu; c KIST Medical College and Teaching Hospital, Lalitpur, Nepal; d M Abdur Rahim Medical College, Dinajpur, Bangladesh

**Keywords:** cardiovascular consequences, Hill-Bone Compliance Scale, hypertension, low-resource setting, Morisky Medication Adherence Scale, Nepal, non-adherence

## Abstract

**Background:**

Nepal is a low resource country with cardiovascular diseases being the number one cause of mortality. Despite hypertension being the single most important risk factor for cardiovascular diseases, non-adherence to anti-hypertensive medications has not been assessed systematically. So, this systematic review and meta-analysis aims to analyze the prevalence of non-adherence to anti-hypertensive medications in Nepal.

**Methodology:**

This systematic review and meta-analysis was piloted in accordance with the Preferred Reporting Items for Systematic Reviews and Meta-Analyses (PRISMA) statement. Electronic databases of Embase, PubMed, Scopus, Web of Science, Cochrane Library, Cinhal Plus, and Google Scholar were searched from inception till 1 February 2023. The random-effects model with 95% confidence interval (CI) was used to calculate the non-adherence rate.

**Results:**

Altogether, 14 studies with a total of 3276 hypertensive patients were included in the meta-analysis. The pooled prevalence of non-adherence to anti-hypertensive medications was 49% (95% CI: 0.37–0.62, *I*^2^=98.41%, *P*<0.001). The non-adherence rate using Morisky Medication Adherence Scale (MMAS) was 55% (95% CI: 0.34–0.76, *I*^2^=99.14%, *P*<0.001), and using Hill–Bone Compliance Scale, the non-adherence rate was 45% (95% CI: 0.37–0.54, *I*^2^=84.36%, *P*<0.001). In subgroup analysis, the non-adherence was higher in rural areas 56% (95% CI: 0.51–0.61, *I*^2^=0.0%, *P*=0.46) compared to urban areas 42% (95% CI: 0.31–0.54, *I*^2^=96.90%, *P*<0.001). The trend of non-adherence was increasing after 2020. Additionally, forgetfulness, carelessness, cost of medications, number of comorbidities, and using an alternate form of medication were common factors associated with non-adherence.

**Conclusions:**

This meta-analysis showed that half of the hypertensive population of Nepal are non-adherent to their anti-hypertensive medications, thereby posing a significant long-term cardiovascular consequence among Nepali population.

## Introduction

Highlights

- Despite the high burden of hypertension (27.3% prevalence), a systematic assessment of non-adherence to anti-hypertensive medications is lacking in Nepal.
- We calculated the pooled prevalence of non-adherence to anti-hypertensive medication in Nepal to be 49% (95% CI: 0.37–0.62, *I*^2^=98.41%, *P*<0.001).
- The difference in non-adherence rate using Morisky Medication Adherence Scale compared to Hill–Bone Compliance Scale was statistically significant (55% vs. 45%; *P*<0.001).
- Rural areas had a higher rate of non-adherence compared to urban areas (56% vs. 42%).
- Common factors associated with non-adherence were forgetfulness, carelessness, cost of medications, number of comorbidities, and using alternate form of medications.

Hypertension (HTN), commonly known as high blood pressure, is a major public health problem worldwide affecting more than 1.28 billion adults^1^. It is estimated that two-thirds of hypertensive patients are from low-income and middle-income countries^1,2^. According to the World Heart Federation, HTN is the number one risk factor for death globally and accounts for about half of all heart disease and stroke-related deaths worldwide^3^.

Optimal management of HTN during the early years is essential to halt morbidity and mortality. Management of HTN is a multidisciplinary approach comprising lifestyle modifications such as healthy diet, exercise, and weight loss in addition to standard anti-hypertensive pharmacological therapy. Compliance with all treatment measures is critical for effective blood pressure management. However, it is reported that up to 50% of hypertensive individuals do not take their medication as prescribed^4^.

A meta-analysis conducted in low-income and middle-income countries estimated that more than 63% of hypertensive patients were non-adherent to the medications^5^. Non-adherence to medication is a multifactorial problem and can be influenced by patient-related factors, such as forgetfulness, fear of side effects, and lack of understanding of the importance of medication. Other factors like drug chosen, the usage of concurrent drugs, the tolerability of the drug, and the duration of drug therapy can also contribute to non-adherence^6^.

Nepal, a low-income country, has a high (27.3%) prevalence of HTN^7^. Due to the high burden of HTN, several studies have been carried out to look at the frequency and causes of non-adherence to anti-hypertensive medicine. However, a systematic pooling of results is lacking. So, this systematic review and meta-analysis aims to evaluate the prevalence of non-adherence to anti-hypertensive medicine in Nepal and to identify risk factors for non-adherence. The findings of this study will assist medical practitioners and policymakers in developing strategies to enhance medication compliance and HTN treatment in Nepal.

## Methodology

### Study protocol

This systematic review and meta-analysis was conducted in accordance with the Preferred Reporting Items for Systematic Review and Meta-Analyses (PRISMA) statement^8^. The PRISMA checklist is presented in Supplementary content (Appendix 1, Supplemental Digital Content 1, http://links.lww.com/MS9/A186). Additionally, we have registered the systematic review in the database of PROSPERO with ID: CRD42023397349. Furthermore, we have reported our manuscript in line with the AMSTAR-2 checklist for systematic review and meta-analysis^9^, details of which are presented in Supplementary content (Appendix 2, Supplemental Digital Content 2, http://links.lww.com/MS9/A187).

### Search strategy

Systematic searches of online databases of Embase, PubMed, Scopus, Web of Science, Cochrane Library, Cinhal Plus, and Google Scholar until 1 February 2023 were conducted to find the relevant articles. The search strategy consisted of Medical Subject Headings (MeSH) terms, keywords, and search terms such as: ‘Hypertension’, ‘blood pressure’, ‘HTN’, ‘medication adherence’, ‘medication compliance’, and ‘Nepal’. Appropriate Boolean operators ‘AND’/‘OR’ were used between the aforementioned terminologies. We did not used any time or language filters during the database search.

As, for example, in the database of PubMed, we used the MeSH terms ‘hypertension’, ‘medication adherence’, and ‘Nepal’. For ‘hypertension’, alternative search terms ‘HTN’ and ‘blood pressure’ were used, all of which were combined with Boolean operator ‘OR’. Similarly, for ‘medication adherence’, alternative search terms ‘medication compliance’ and ‘adherence’ were combined with Boolean operator ‘OR’. Finally, all search terms were combined with the Boolean operator ‘AND’, which resulted in the final search results of PubMed. The details of the search strategy are shown in the Supplementary content (Appendix 3, Supplemental Digital Content 3, http://links.lww.com/MS9/A188).

### Selection criteria

#### Inclusion criteria

Cross-sectional studies conducted either in hospital or community-based setting analyzing medication adherence among hypertensive patients of Nepal from inception were included in the systematic review. We did not take into consideration the particular type of scale used for assessing medication adherence provided the scale was internationally acceptable and previously used in similar settings.

#### Exclusion criteria

1. Studies assessing medication adherence as yes/no response in hypertensive patients.
2. Studies where medication adherence has been assessed after a type of intervention such as health education, awareness programs, and pharmacist counseling.
3. Case reports/case series, review articles, correspondences, narratives, poster presentation costs simulation studies, and studies where our outcomes of interest seemed lacking were excluded from the review.
4. Studies with irretrievable full text.

### Study selection

Two authors systematically searched the databases for relevant articles. First, the title of individual studies were screened to find the duplicates. After sorting out duplicate articles, remaining articles were screened by reading the title and abstract. Following this, the full texts of the relevant articles at this point were selected for the systematic review based on the aforementioned inclusion and exclusion criteria. Additionally, bibliographies of selected articles were also searched to identify relevant studies. The final list of included studies had the concurrence of all authors.

### Data extraction

Studies obtained from the electronic databases, supplementary sources, and manual searching were exported to Endnote reference software version 20.2 (Thomson Reuters, Stamford, Connecticut, USA) in a compatible format. Duplicate articles were screened first by Endnote and then manually. Duplicates were then recorded and removed. For multiple publications of the same data in more than one journal, the most inclusive, comprehensive studies, with larger sample size, and the most recent ones were considered. Basic data extraction was conducted in Microsoft Excel spreadsheet.

### Data items

All authors had agreed on the set of predefined data items to be extracted from the selected articles. Based on this, two authors independently extracted the studies for data items. The data items extracted included author, year, province/district of study conducted, sampling technique, sample size, male/female, age (mean/median/range), study setting (hospital vs. community based, urban vs. rural), tools utilized to assess medication adherence, number of adherents/non-adherent patients, and percentage of non-adherence. For studies reporting adherence as low, moderate, and high degree, both low and moderate degrees of adherence cases were considered as non-adherent to hypertensive medication. Furthermore, if applicable, factors associated with medication non-adherence were extracted.

Authors of some studies were contacted via E-mail and/or ResearchGate for retrieval of full text and clarification of doubts wherever required. Any misunderstanding created during the data extraction process was sorted by making a common consensus between the authors. Additionally, the data extracted by the primary authors was cross-checked by a third author independently.

### Tools for measuring medication adherence

#### Morisky Medication Adherence Scale (MMAS)

In 1986, MMAS was developed originally as a four-item scale (MMAS-4) using four questions with dichotomous response categories ‘yes’ or ‘no’^10^. The scale is scored by one point for each ‘no’ response and zero points for each ‘yes’ response, with the total score ranging from zero to four. MMAS-4 is also called Morisky Green scale. In 2008, a modified eight-item scale (MMAS-8) was published^11^. The first seven items of MMAS-8 have a dichotomous response category with ‘yes’ or ‘no’, and the last item uses a five-point Likert response scale. The four items added in the modified version aim to identify and address the circumstances or situations related to the adherence of the patient. The score ranges from zero to eight, with higher scores implying a higher degree of adherence. If the total score is less than six, the patient is considered non-adherent. We considered both MMAS-4 and MMAS-8 in the meta-analysis.

#### Hill–Bone Compliance Scale (HBCS)

The HBCS focuses on adherence to anti-HTN medication^12^. The scale assesses patient behavior by including three behavioral subscales: reduced sodium intake, appointment timeliness, and medication intake. The scale has 14 items within the three subscales. Each item has a four-point Likert scale. Higher the score, higher is the degree of adherence.

#### Brief Medication Questionnaire (BMQ)

The BMQ explores both the patient’s medication-taking behavior and barriers to adherence^13^. It consists of three different screens; a five-item regime screen, a two-item belief screen, and a two-item recall screen. These screens assess how patients took each of their medications in the past week, with drug efficacy and bothersome features and remembering difficulties, respectively.

#### Modified Treatment Adherence Questionnaire for Patients with Hypertension (MTAQPH)

This questionnaire was made by modifying the Treatment Adherence Questionnaire for Patients with Hypertension (TAQPH) by Ma *et al.* ^14^. Altogether, the scale consists of 41 items and a score ranging from 41 to 164. Higher score signifies the higher treatment adherence.

#### Self-reported compliance test

Shrestha *et al.* ^15^ made a three-item questionnaire for assessing medication adherence, adapting the validated questionnaires from the literature.

### Quality assessment

The quality assessment of the included studies was conducted using the Newcastle–Ottawa scale adapted for observational studies^16^. The scale consists of three components: selection (maximum 5 stars), comparability (maximum 2 stars), and outcome (maximum 3 stars). The risk of bias was stratified into low risk (score >7), intermediate risk (score 4–7), and high risk (score <4) based on the score each study gets during the quality assessment. Only studies with low and intermediate risk of bias were included in the meta-analysis. The details of quality assessment are presented in the Supplementary content (Appendix 4, Supplemental Digital Content 3, http://links.lww.com/MS9/A188).

### Statistical analysis

All basic calculations were performed in Microsoft Excel 2016 (Microsoft Corp., Redmond, Washington, USA). For further analysis, data from the Excel sheet were extracted using STATA version 17.0 (Stata Corp., College Station, Texas, USA). Pooling of non-adherence to anti-hypertensive medications was done with DerSimonian and Laird’s^17^ random-effects model due to different population and demographic settings across studies. With this difference, we anticipated considerable heterogeneity among the studies. Cochrane Q test and *I*^2^ statistics were used to examine the heterogeneity between studies. Substantial heterogeneity was measured for a value of *I*^2^ higher than 75%^18^. All analysis with two-tailed with a significance level set to less than 0.05.

For the determination of the source of heterogeneity, further subgroup analysis was done for: type of scale used for assessing medication adherence, rural vs. urban study setting, and study period (before 2020 and after 2020). Additionally, sensitivity or leave-one-out analysis was done to check the influence of the individual studies on the overall study analysis. Publication bias was evaluated by the funnel plot of the overall effect size with standard error (SE). For the small study effect size, Egger’s regression test was performed. *P* value less than 0.1 was regarded statistically significant for publication bias.

## Results

### Study selection

**Figure 1:**
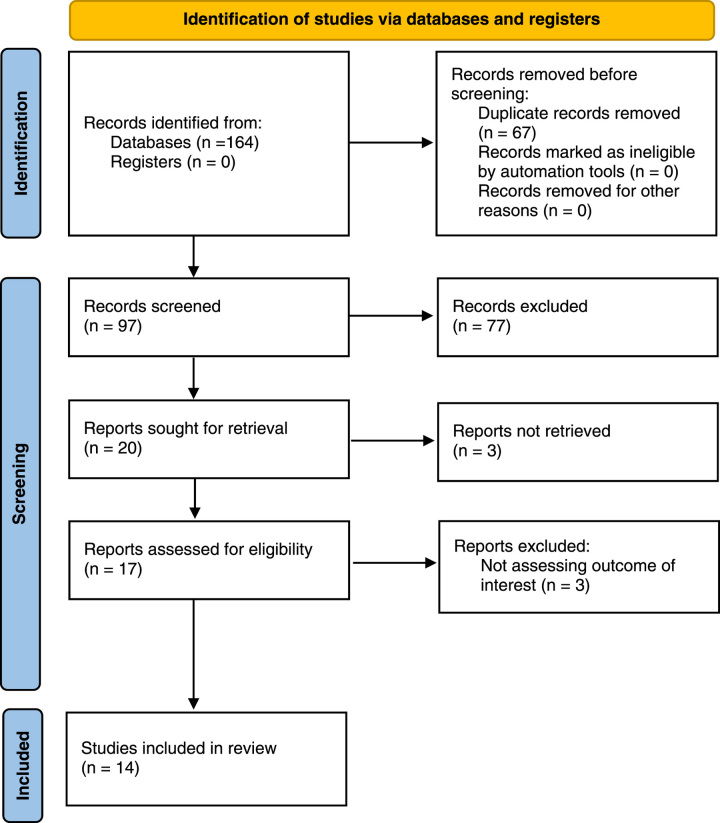
PRISMA (Preferred Reporting Items for Systematic Reviews and Meta-Analyses) diagram showing study selection process.

Altogether, 164 articles were obtained from the databases of Embase, PubMed, Scopus, Web of Science, Cochrane Library, Cinhal Plus, and Google Scholar, from inception till 1 February 2023. From the initial search results, 67 duplicate articles were removed. From the remaining 97 articles, 77 articles were removed by screening the title and abstract. Full-text review was done thoroughly in the remaining 20 articles, out of which six articles were excluded based on the eligibility criteria. Finally, 14 full-text articles were included for the analysis. The PRISMA diagram tailoring the details of the study selection process is shown in Figure 1.

### Study characteristics

**Figure 2:**
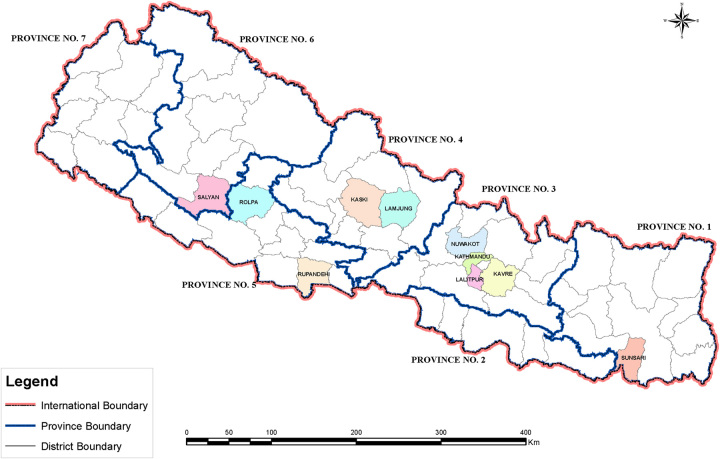
Map of Nepal with districts where studies were conducted.

These 14 studies included a total of 3276 hypertensive patients, with the sample size ranging from 79 to 673. Two studies had not reported male and female patient proportions. However, in the remaining 12 studies, the number of male and female patients were 1424 and 1507, respectively, with the male-to-female ratio being 0.95. Among the 14 studies, seven were conducted in a single district, Kathmandu. Following this, two studies were from Rupandehi, two from Kavre, one from Sunsari, Nuwakot, Lamjung, Rolpa, Lalitpur, Kaski, and Salyan districts respectively. The map of Nepal with the area of conduction of the included studies is shown in Figure 2. The studies of Bhandari *et al*.^19^, Devkota *et al*.^20^, and Khan *et al*.^21^ were conducted in community-based settings, whereas the remaining 11 studies were hospital based. Similarly, two studies by Shakya *et al*.^22^ and Sharma *et al*.^23^ were conducted in rural settings. The studies of Rana *et al*.^24^ and Khadka *et al*.^25^ were conducted in rural as well as urban settings. The remaining 10 studies were conducted in urban settings.

For assessing medication adherence, Khan *et al*.^21^ used the BMQ and Maharjan^26^ used the MTAQPH. The HBCS was utilized by Bhusal *et al*.^27^, Shakya *et al*. 2020^28^, Shakya *et al* 2022^22^ and Shrestha *et al*.^29^. Similarly, Shrestha *et al*.^15^ used the self-made questionnaire adapting the tools of Morisky–Green and Wong. Remaining seven studies, that is the studies of Rana *et al*.^24^, Bhandari *et al*.^19^, Devkota *et al.* ^20^, Khadka *et al.* 2017^30^, Khadka *et al.* 2021^25^, Roka *et al.* ^31^ and Sharma *et al.* ^23^ used the MMAS. In these 14 studies, a total of 1634 patients were non-adherent to anti-hypertensive medications. The percentage of non-adherence ranged from 15.3% in the study of Maharjan^26^ to 80.5% in the study of Rana *et al*.^24^. The detail baseline characteristics of the included studies are presented in Table 1.

**Table 1:** Baseline characteristics of the included studies

Author	Year of publication	District of study conducted	Sampling technique	Sample size	Male	Female	Age (mean/median/range) years	Study setting (hospital vs. community based)	Study site (urban vs. rural)	Tools utilized to assess medical adherence	Number of adherent patients	Number of non-adherent patients	Percentage of non-adherence
Rana *et al*.^24^	2020	Rupandehi (Bhairahawa)	Convenience sampling	384	168	216	57.04±10.03	Hospital based	Urban and rural	Morisky Medication Adherence Scale	75	309	80.5
Bhandari *et al*.^19^	2015	Sunsari (Dharan)	Random sampling	154	NA	NA	NA	Community based	Urban	Morisky Medication Adherence Scale	87	67	43.5
Bhusal *et al*.^27^	2022	Kathmandu	Non-probability consecutive sampling	150	80	70	NA	Hospital based	Urban	Hill–Bone Compliance Scale	94	56	37.33
Devkota *et al*.^20^	2016	Kathmandu	Multistage stratified sampling	191	NA	NA	NA	Community based	Urban	Morisky Medication Adherence Scale	38	55	59.14
Khadka *et al*.^30^	2017	Rupandehi (Bhairahawa)	Random sampling	673	289	384	52.90±16.37	Hospital based	Urban	Morisky Medication Adherence Scale	512	161	23.92
Khadka *et al*.^25^	2021	Nuwakot, Lamjung, Rolpa, Kathmandu, Lalitpur	Convenience sampling	348	183	165	NA	Hospital based	Rural and urban	Morisky Medication Adherence Scale	83	265	76.1
Khan *et al*.^21^	2013	Kaski (Pokhara)	Random sampling	79	44	35	NA	Community based	Urban	Brief Medication Questionnaire	28	51	64.6
Maharjan *et al*.^26^	2016	Kathmandu	Purposive sampling	85	53	32	51.81 (SD=11.38)	Hospital based	Urban	Modified Treatment Adherence Questionnaire for Patients with Hypertension	72	13	15.3
Roka and Ghimire^31^	2019	Kathmandu	Sequential sampling	216	110	106	NA	Hospital based	Urban	Morisky Medication Adherence Scale	60	156	72.2
Shakya *et al*.^28^	2020	Kathmandu	Non-probability purposive sampling	204	104	100	60±12	Hospital based	Urban	Hill–Bone Compliance Scale	101	103	50.5
Shakya *et al*.^22^	2022	Kathmandu	Convenience sampling	282	148	134	58.49±12.44	Hospital based	Rural	Hill–Bone Compliance Scale	127	155	55
Sharma *et al*.^23^	2021	Salyan	Random Sampling	110	36	74	NA	Hospital based	Rural	Morisky Medication Adherence Scale	45	65	59.1
Shrestha *et al*.^15^	2018	Kavre (Dhulikhel)	Random sampling	260	132	128	NA	Hospital based	Urban	Self-Reported Compliance Test	135	125	48.1
Shrestha *et al*.^29^	2019	Kavre	Convenience sampling	140	77	63	53.82 (SD=12.12)	Hospital based	Urban	Hill–Bone Compliance Scale	87	53	37.9

### Quality assessment

The Newcastle–Ottawa scale was used as a checklist to assess the quality of the included studies. Among the 14 studies, only the study of Devkota *et al*. had a low risk of bias (mean score 7.5). The remaining 13 studies had an intermediate risk of bias (mean score 4–7). So, all of the 14 studies were included in the final meta-analysis. The details of quality assessment are presented in the Supplementary content (Appendix 4, Supplemental Digital Content 3, http://links.lww.com/MS9/A188).

### Meta-analysis

#### Pooled prevalence of non-adherence

**Figure 3:**
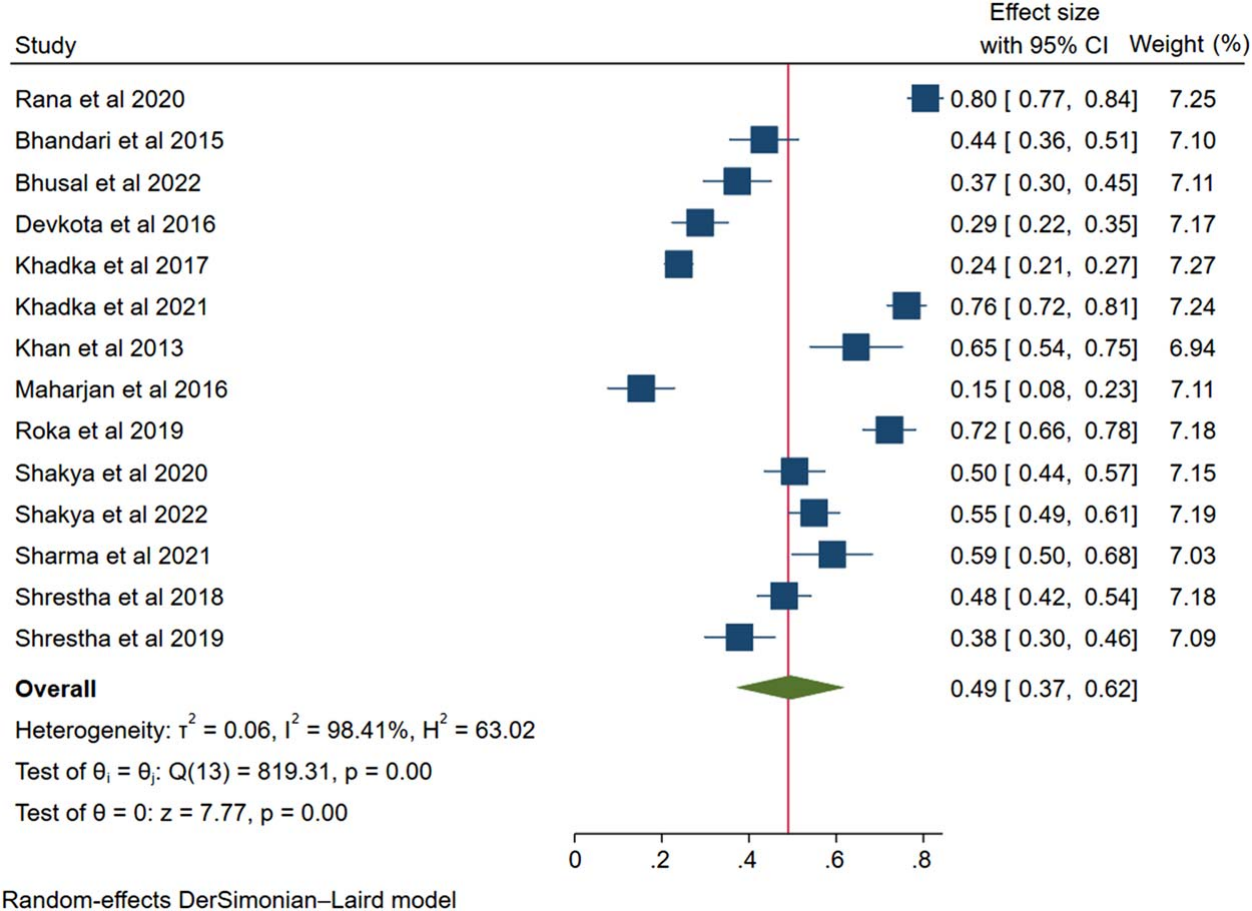
Forest plot showing the pooled prevalence of non-adherence.

Using the random-effect DerSimonian Laird method, the pooled prevalence of non-adherence to anti-hypertensive medications among the Nepali hypertensive patients was 49% (95% confidence interval (CI): 0.37–0.62, *I*^2^=98.41%, *P*<0.001). This is shown in the forest plot image of Figure 3.

#### Subgroup analysis

As the degree of heterogeneity was substantial in the overall pooled prevalence, further subgroup analysis was conducted for: tools used for assessing medication adherence, study site (rural vs. urban, Kathmandu vs. outside Kathmandu), and trend analysis (before 2020 vs. after 2020).

*(A) Non-adherence prevalence based on the tool used for assessing medication adherence*: Among the tools utilized, seven studies had used the MMAS, whereas four studies had used HBCS. The remaining three studies had used individual separate tools for assessing medication adherence.

**Figure 4:**
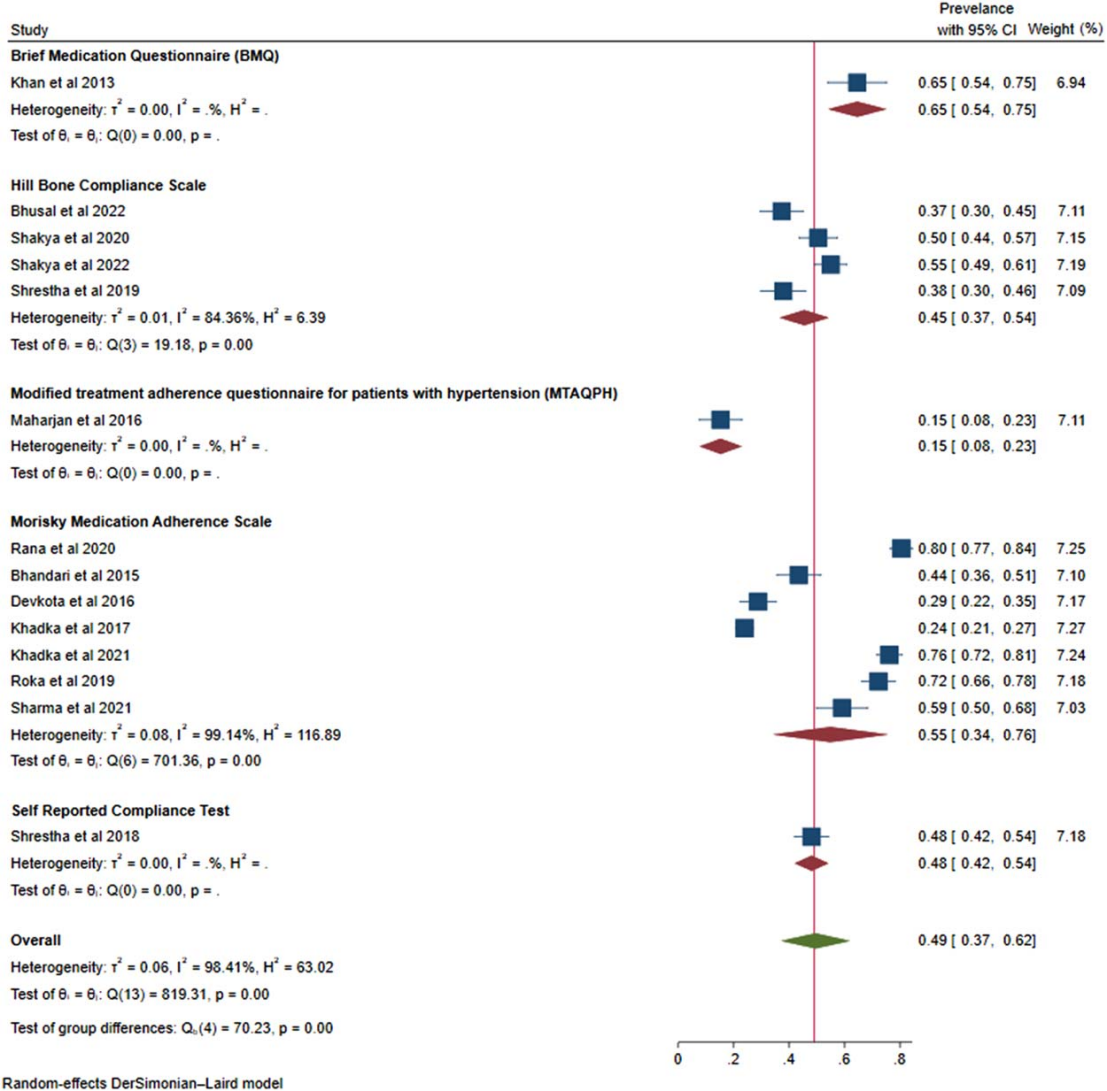
Forest plot showing prevalence of non-adherence according to the tool used.

The prevalence of non-adherence by using MMAS was 55% (95% CI: 0.34–0.76, *I*^2^=99.14%, *P*<0.001), and HBCS 45% (95% CI: 0.37–0.54, *I*^2^=84.36%, *P*<0.001). The details are presented in the forest plot image of Figure 4.

**Figure 5:**
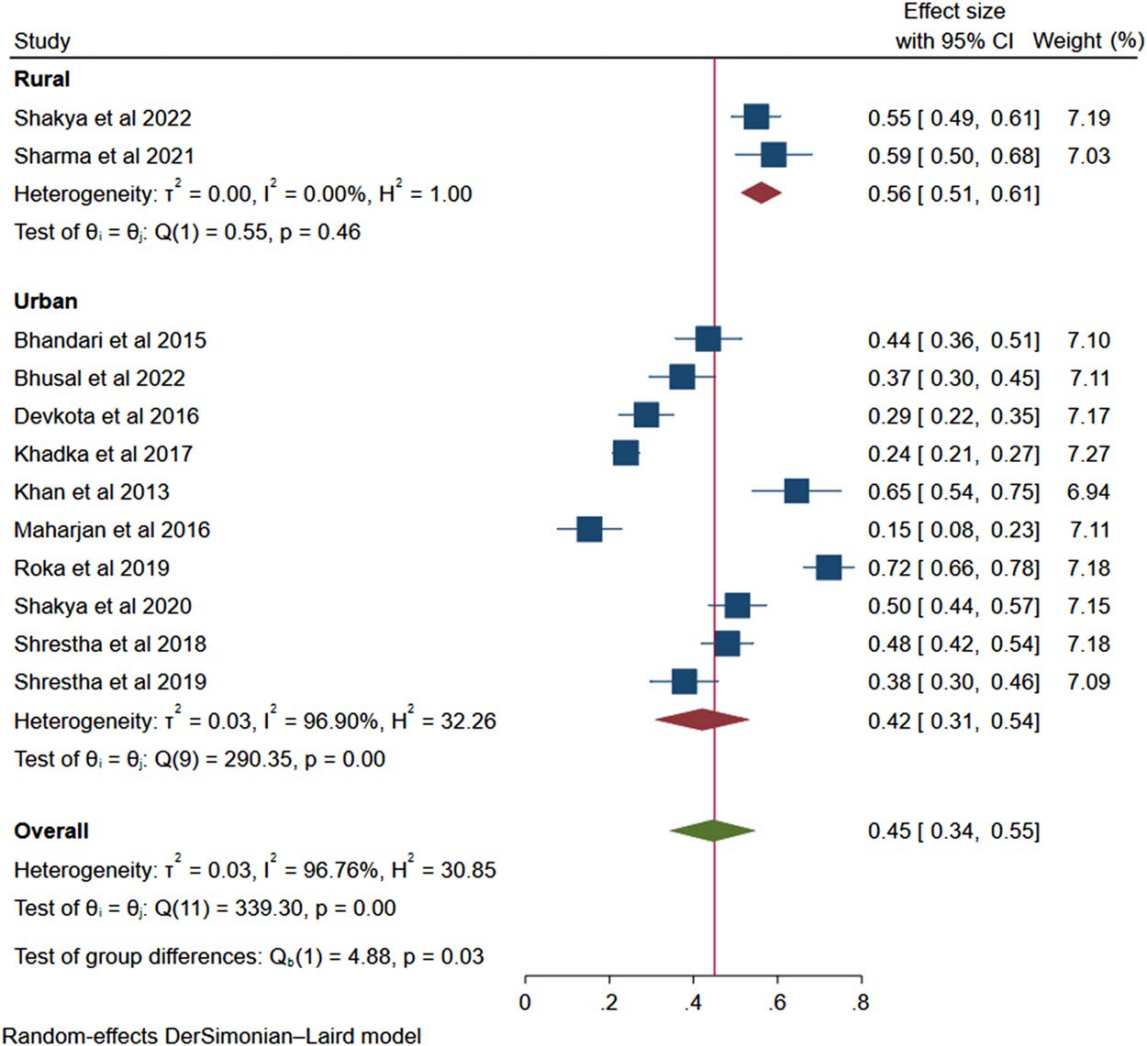
Forest plot showing prevalence of non-adherence in rural and urban settings.

*(B) Study site*: The prevalence of non-adherence in rural areas was higher 56% (95% CI: 0.51–0.61, *I*^2^=0.0%, *P*=0.46) compared to urban areas with a non-adherence prevalence of 42% (95% CI: 0.31–0.54, *I*^2^=96.90%, *P*<0.001). The higher non-adherence rate in rural areas was statistically higher than the non-adherence rate in urban areas (*P*<0.001). This is shown in the forest plot image of Figure 5.

Similarly, the prevalence of non-adherence within Kathmandu was 43% (95% CI: 0.27–0.60, *I*^2^=97.21%, *P*<0.001) compared to outside Kathmandu 54% (95% CI: 0.36–0.73, *I*^2^=98.88%, *P*<0.001). This is illustrated in the Forest plot image of Supplementary content (Appendix 5, Supplemental Digital Content 3, http://links.lww.com/MS9/A188).

**Figure 6:**
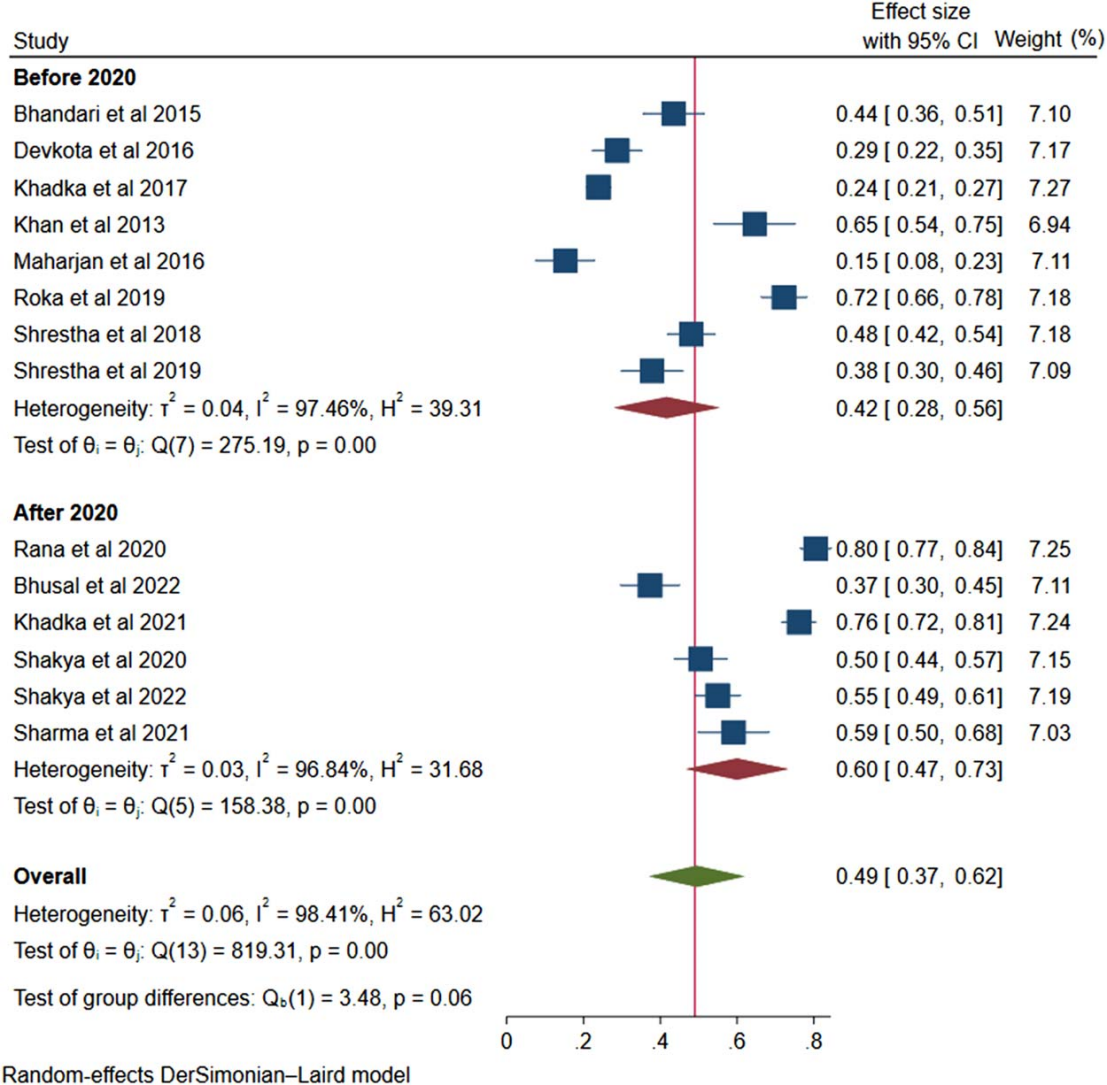
Forest plot showing trend of non-adherence before 2020 and after 2020.

*(C) Trend of non-adherence*: The prevalence of non-adherence before the year 2020 was 42% (95% CI: 0.28–0.56, *I*^2^=97.46%, *P*<0.001). Similarly, the non-adherence prevalence after 2020 was 60% (95% CI: 0.47–0.73, *I*^2^=96.84%, *P*<0.001). The trend has been presented in the forest plot image of Figure 6.

#### Sensitivity analysis

No significant changes were obtained in the overall results and the degree of heterogeneity when the studies were omitted one by one, thus establishing the robustness of meta-analysis. The details of the sensitivity analysis are presented in the Supplementary content (Appendix 6, Supplemental Digital Content 3, http://links.lww.com/MS9/A188).

#### Publication bias

The funnel plot for publication bias was relatively symmetrical. Moreover, regression-based Egger’s test (*P*=0.75) and Begg’s test (*P*=0.66) did not show an indication of a small study effect, confirming no evidence of publication bias in the meta-analysis. Details of the publication bias analysis are shown in the Supplementary content (Appendix 7, Supplemental Digital Content 3, http://links.lww.com/MS9/A188).

### Factors associated with non-adherence

Not all studies had reported the factors associated with non-adherence to anti-hypertensive medications. Among the reported studies, forgetfulness, carelessness, cost of medications, number of comorbidities, and using an alternate form of medications were the common factors associated with medication non-adherence. Except in the study of Bhandari *et al*. and Shrestha *et al*., factors were not reported as odds ratio/relative risk ratio, so meta-analysis could not be conducted. The details of factors associated with non-adherence are presented in Table 2.

**Table 2:** Factors associated with non-adherence to anti-hypertensive medications

Study	Year	Factors associated with non-adherence/adherence
Bhandari *et al.* ^19^	2015	Illiteracy, uncontrolled blood pressure, expensive price of medicine, no family history of hypertension, irregular follow-up, and more than one pill per day
Bhusal *et al.* ^27^	2022	Forgetfulness, ineffective counseling, missed follow-up, number of pills
Devkota *et al.* ^20^	2016	Thinking they completely recovered from the illness, reducing the dose themselves when they felt well
Khadka *et al.* ^25^	2021	Forgetfulness, expensive medication, use of Ayurveda and other forms of medicine, regular exercise and low-salt diet, fear of addiction to medicine, no information regarding complications, difficulty for regular follow-up
Shrestha *et al.* ^29^	2019	Economic status, patient doctor relationship, knowledge about hypertension, social support
Maharjan *et al.* ^26^	2016	Knowledge on hypertension and its consequences, previous control of hypertension with medications, emotions
Rana *et al.* ^24^	2020	Sex, living in rented house, years of hypertension
Roka and Ghimire^31^	2019	Sex, comorbidities, getting medication free of cost
Shrestha *et al.* ^15^	2018	Age, sex, living in rented house, low socioeconomic status, married status, employment status, distance to the drug store/pharmacy

## Discussion

This review is the first of its kind to systemically assess the scientific literature on non-adherence to anti-hypertensive medications in a lower-middle-income country, Nepal. From the meta-analysis, the prevalence of non-adherence to anti-hypertensive medications among the Nepali hypertensive patients was 49% (95% CI: 0.37–0.62, *I*^2^=98.41%, *P*<0.001).

On comparing with regional rates, the non-adherence to anti-hypertensive therapy in South Asia was 48%, East Asia 45%, and the Middle East 41%^32^. Similarly, a higher rate of non-adherence was observed in low and lower-middle-income countries, that is 50% compared to upper-middle and high-income countries, that is 37% and 44%, respectively^32^. Moreover, in the neighboring countries, the non-adherence rates are: India (43%), Bangladesh (58%), and Pakistan (45%)^32^. This shows a high degree of non-adherence among South Asian countries including Nepal.

Numerous factors, such as forgetfulness, adverse effects, cost, ignorance, and trust in alternative treatments, have contributed significantly to the rise in anti-hypertensive drug non-adherence. Forgetting is a common cause of non-adherence, particularly if they are taking many drugs or have complicated dosage schedules. Some patients may experience anti-hypertensive drug side effects that make it challenging to stick to their medication^4,27^. People may not be aware of the consequences of a missing a dose or understand how medication works to control HTN. Some people might decide against using conventional medicine in favor of alternative treatments or lifestyle changes like exercising or changing their diet^4^. These steps may be useful, but they may not be sufficient in and of themselves to control HTN. Additionally, lack of proper basic counseling regarding the pattern of medication use is responsible for low adherence^25,27^. Lack of awareness on the pathophysiology of HTN and the need to take medications lifelong is also responsible for low adherence^33^.

From the subgroup analysis, the non-adherence rate in rural areas (56%) was higher than in the urban areas (42%). In the context of Nepal, rural areas have always been backward in all relevant healthcare-related sectors and development. The need for constant follow-up, difficulty associated with timely getting access to the required level of healthcare due to diverse and arduous topography, lower level of literacy, and low HTN awareness could be the possible reasons resulting in such increased non-adherence. Additionally, non-adherence was lower in Kathmandu (43%) compared to outside Kathmandu (54%). This is because of the easy access of health resources, higher literacy, and higher patient awareness in Kathmandu. Previous study has also shown better adherence among urban group of population compared to rural population^34^. Furthermore, the non-adherence rate was significantly variable within the same district. This was because of the variability in the use of the tool for measuring non-adherence. Second, within the same district, there were studies from urban areas as well as rural areas, resulting in significant variation in non-adherence rate. Additionally, the community-based vs. hospital-based study setting had some impact on the non-adherence rates.

Assessing medication adherence is crucial in controlling blood pressure. Various medication adherence tools have been used in Nepal, namely, Morisky Medication Adherence Scale (MMAS), Hill–Bone Compliance Scale (HBCS), Brief Medication Questionnaire (BMQ), and Modified Treatment Adherence Questionnaire for Patients with Hypertension (MTAQPH). Among these tools, only HBCS has been translated in Nepali language and validated in Nepal^22^. Thus, it is simple and easy to use in a resource-limited setting like Nepal and ensures cultural appropriateness and validity in the local context. In this meta-analysis, the prevalence of non-adherence using MMAS was higher (55%) compared to HBCS (45%). Furthermore, the HBCS is specific for assessing non-adherence in hypertensive patients, making it a more valid tool and with less bias^12^. In MMAS, questions are designed to take advantage of the ‘yes-saying’ bias in order to elicit disclosure of non-adherence, so the non-adherence rate is likely to be high^10,11^.

From the subgroup analysis, non-adherence rate is in an increasing trend, that is more after 2020 compared to before 2020. Emergence of COVID-19 pandemic might have a role in decreasing anti-hypertensive drug adherence after 2020. People’s everyday lives have seen major upheaval as a result of the COVID-19 pandemic, including interruptions to the healthcare system and pharmaceutical access^35^. There may have been several missed opportunities to see their doctors, which might have resulted in lost opportunities for prescription refills and increased non-adherence. The pandemic’s economic effects have been felt by many, and people’s capacity to pay for their prescriptions has been hampered by job losses and financial strain. Anxiety and stress levels have grown as a result of the pandemic, which may make it harder for patients to take their medications as prescribed. Moreover, stress and worry can raise blood pressure, which makes it even more crucial to follow prescription instructions.

Chronic diseases such as HTN for decades have always been a major public health concern among developing nations. Maintenance of drug adherence in lower-middle-income countries is always arduous, resulting in increased morbidity and mortality. A study carried out by Dhungana *et al*.^33^ in Nepal revealed that the prevalence of HTN screening, awareness, treatment, and control was 65.9%, 20%, 10.3%, and 3.8%, respectively. So there is a substantial danger of long-term cardiovascular sequelae in a nation with very poor levels of awareness of hypertension and therapy, with half of the patients not adhering to medication.

Anti-hypertensive drug non-adherence can be decreased using a variety of techniques. Fewer prescriptions, simplified dosage instructions, pillboxes, or reminders can be used in pharmaceutical regimens to make it easier for patients to follow their drug schedules and reduce forgetfulness^27^. Healthcare professionals should discuss the mechanism of medication, appropriate use of medications, possible side effects, and their management considering patient literacy level^36^. Moreover, patients and medical staff can work together to adjust dosages or switch to medications with fewer side effects. Moreover, it is crucial to make sure that patients are aware of the possible consequences of not taking their medicine on their health and finances^36^. Providers can help patients manage the cost of medicines by helping them find potential sources of financial support, such as patient assistance programs or generic drug options. Patients who exhibit a belief in alternative therapies can be helped by providers to comprehend the advantages of conventional medicine. It is crucial to acknowledge the possible advantages of lifestyle modifications while simultaneously highlighting the significance of drug adherence for managing HTN. Clinicians should be aware of how patients’ adherence to their medications is being impacted by the COVID-19 epidemic and work with them to eliminate any barriers to care, such as missed appointments, a backlog of prescription refills, or concerns about contracting the virus during in-person visits. Telehealth services may also be considered in order to improve access to care.

Non-adherence to treatment is also common in other chronic diseases in Nepal. Study done by Shrestha *et al*.^37^ showed that more than 52% of chronic obstructive pulmonary disease patients were non-adherent to their medications. Another study of Sah *et al*.^38^ reported that more than 65% of type II diabetes mellitus patients were non-adherent to their anti-hyperglycemic medications. Such data of high non-adherence to treatment in non-communicable diseases (NCDs) suggests the utmost need for proper public health planning and urgent plan of action to bring such medication non-adherence under control in the near future. The Government should implement a combined approach plan to address the low level of awareness, treatment, and adherence to the major NCDs and their risk factors.

Our study has several strengths. First, this is the only study to assess the medication non-adherence among hypertensive patients of Nepal systematically. Second, we have addressed the common factors associated with non-adherence. Third, we have done subgroup analysis to address the source of heterogeneity. Fourth, the sensitivity analysis did not show an individual study effect on the overall results, and there was no evidence of publication bias. Despite this, this study was not free of limitations. First, the heterogeneity was considerable despite the subgroup analysis. Second, meta-analysis of the factors associated could not be conducted because of scarce reporting of data in a measurable form, and the unavailability of other types of randomized and non-randomized studies to look over these aspects. Third, additional subgroup analysis for age, sex, and comorbidities could not be conducted. Therefore, the readers of this meta-analysis should consider these shortcomings while making a decision.

As there is a paucity of data on HTN and its treatment from different parts of the country, our research opens the door for new researches addressing these gaps. In Nepal, there is seemingly a lack of proportionate distribution of anti-hypertensive medications, and the cost of available medications is high. This has resulted in high out-of-pocket expenses resulting in higher non-adherence rates. This problem needs to be addressed by the Government and stakeholders in order to reduce the HTN-related morbidity and mortality. Furthermore, our research showed that low levels of awareness and forgetfulness were associated with high non-adherence to medications. In order to tackle these issues, awareness of grassroot levels should be increased so that people will realize that having untreated HTN is the single most important risk factor for cardiovascular sequelae.

## Conclusions

The pooled prevalence of non-adherence to anti-hypertensive medication in Nepal was 49%. Using MMAS, the non-adherence prevalence was 55%, and non-adherence prevalence using HBCS was 45%. Additionally, the non-adherence rate was higher in rural areas (56%) compared to urban areas (42%). Common factors associated with non-adherence were forgetfulness, carelessness, cost of medications, number of comorbidities, and using an alternate form of medications. As half of the hypertensive patients are non-adherent to their medications in Nepal, we recommend the key stakeholders to formulate a common action plan to address this serious issue to decrease the long-term cardiovascular consequences.

## Ethical approval

Ethical approval is not required for our study.

## Consent

Consent is not required for our study.

## Sources of funding

None.

## Author contribution

P.P.: conceptualization, methodology, data curation, data analysis, writing – original draft, writing – review and editing, project administration; S.K.Y., A.A., S.K., and N.P.: data curation, methodology, writing – original draft; A.B.S.: statistical analysis, writing – original draft; S.G.: writing – original draft; writing – review and editing. All authors have read and agree to the final version of the manuscript.

## Conflicts of interest disclosure

The authors declare that there are no conflicts of interest.

## Research registration unique identifying number (UIN)

None.

## Guarantor

Pashupati Pokharel, ORCID: 0000-0002-9704-5883.

## Data availability statement

The data supporting the findings of the manuscript will be made available upon considerable request to the corresponding author.

## Provenance and peer review

Not commissioned, externally peer-reviewed.
